# LiPISC: A Lightweight and Flexible Method for Privacy-Aware Intersection Set Computation

**DOI:** 10.1371/journal.pone.0157752

**Published:** 2016-06-21

**Authors:** Wei Ren, Shiyong Huang, Yi Ren, Kim-Kwang Raymond Choo

**Affiliations:** 1 School of Computer Science, China University of Geosciences, Wuhan, Hubei, P.R. China; 2 Hubei Key Laboratory of Intelligent Geo-Information Processing (China University of Geosciences (Wuhan)), Wuhan, Hubei, P.R. China; 3 Department of Computer Science, National Chiao Tung University, Hsinchu, Taiwan, R.O. China; 4 School of Information Technology and Mathematical Sciences, University of South Australia, Adelaide, South Australia, Australia; King Saud University, Kingdom of Saudi Arabia, SAUDI ARABIA

## Abstract

Privacy-aware intersection set computation (PISC) can be modeled as secure multi-party computation. The basic idea is to compute the intersection of input sets without leaking privacy. Furthermore, PISC should be sufficiently flexible to recommend approximate intersection items. In this paper, we reveal two previously unpublished attacks against PISC, which can be used to reveal and link one input set to another input set, resulting in privacy leakage. We coin these as Set Linkage Attack and Set Reveal Attack. We then present a lightweight and flexible PISC scheme (LiPISC) and prove its security (including against Set Linkage Attack and Set Reveal Attack).

## 1 Introduction

Online social networks (including enterprise social networks such as Yammer) and e-commerce websites are increasingly popular with both individual and organizational users. In these applications, recommendations are a frequently used mechanism for users and service providers to suggest potential friends or interested commodities to other users. For example, in online social networks such as Tencent QQ or Facebook, service providers recommend potential friends to another user, by relying on the intersection set of the user’s friends’ friends. More specifically in this context, B and C are A’s friends; thus, the intersection set of B’s friends and C’s friends may be A’s potential friends. The service provider needs to conduct regular intersection set computation (ISC) to find the set D that excludes A’s friends. Users in set D are then recommended to A as potential friends. Similarly, services or other contacts (e.g., prospective partners for dating apps) are recommended using this (recommendation computation) approach.

Recommendation computation can be generalized as the calculation of an intersection function, where the input is two or more sets and the output is the intersection set of the input sets. We remark that the entity (e.g., online social network service providers) for computing intersection function may not always be trustworthy. Hence, the privacy of users may be leaked (or compromised) during the computations. Therefore, privacy-aware computation of intersection set has attracted the attention of security and privacy researchers (see [1–7]), and is key to the widespread adoption of online social networks and e-commence websites.

Most existing studies address specific privacy protection problems in the context of a particular application. Relatively few research formalize the privacy-aware computation of intersection set problem as an abstract problem (i.e., privacy-aware intersection set computation (PISC)) or focus on designing schemes that are lightweight and flexible. This is the gap that we seek to address in this paper. We also reveal two previously published security attacks against PISC, namely: set reveal attack and set linkage attack.

In this paper, we propose a lightweight and flexible PISC (hereafter referred to as LiPISC) scheme, which satisfies the following properties:

1. A basic requirement for privacy protection is for intersection computation to be conducted over input sets with concealed members instead of plain members. This is more difficult than traditional ISC.
2. An enhanced requirement for privacy protection is unlinkable. In other words, computation entities cannot infer the existence of the same items using the received input sets after two computation operations.
3. A basic requirement for flexibility is the capability to compute rough set. That is, PISC can return approximate intersection set even though matched common members do not exist in input sets.
4. Lightweight and scalable for large scale applications.
5. Security against set reveal attack, set linkage attack, and other common attacks.

We then prove the security of LiPISC.

The remainder of this paper is organized as follows. Section 2 describes related work. In Section 3, we present the basic assumption and models used in the paper. Section 4 details our proposed LiPISC scheme and the security proof. Finally, Section 5 concludes this paper.

## 2 Related Work

Ensuring the privacy of recommendations in social networks is a current research focus (see [8–15]). Wicker and Schrader [8] surveyed the philosophical, legal, moral, and epistemological literature on privacy in information networks, and introduced privacy-aware design principles. In the same year, Pentafronimos, Karantjias and Polemi [9] identified privacy requirements in collaborative workspaces, and suggested a number of guidelines for privacy-aware identity and access management systems. Attempting to fulfill the privacy requirements in online social networks, Akcora, Carminati and Ferrari [10] proposed a privacy risk measure to provide users a way of estimating risk (via the use of a Facebook prototype application). In 2013, Shoaran, Thomo and Weber-Jahnke [11] studied privacy issue in big data. They used graphs to model big data and proposed privacy-aware release of graph summarization using zero-knowledge privacy. Similarly, Vidyalakshmi et al [12] proposed a privacy aware information dispersal method in social networks, where they employed a supervised learning model to assist user in spotting unintended audience for a post. A year later in 2014, Li [13] posited that users should be able to indicate different comfort levels to share their friendships in social networks, and this can be done by limiting the number of their friends returned in response to queries through the friend search engine. They also proposed a new attack model that may infer more friendship information based on the friendships detected in the query results, as well as defining a model to safely display friends of individual users in response to queries. However, these studies only focused on specific applications, rather than seeking to solve the underlying challenge using a generalized approach.

Privacy protection of recommendations in e-commerce systems has also been the subject of extensive research. For example, Bunea et al [16] presented a privacy-aware collaborative filtering recommender framework, where they used a probabilistic matrix factorization technique to mitigate the sparsity and a dynamic privacy inference model to ensure privacy. In the dynamically personalized recommendation algorithm of Tang and Zhou [17], information in ratings and profile contents were used, without a strong focus on privacy protection. He, Ren and Zhang [18] presented an approach to increase the conversion rate from browsing to buying. Their method uses a behavior-based inference of a customer’s propensity to purchase from a product category. Celdran et al [19] proposed a middle-ware to provide users with custom context-aware recommendations. However, these studies neither focused on privacy protection nor presented a more general method that can be deployed in a wide range of applications. We also observe that there is no suggestion that PISC can be modeled as secure multi-party computation.

PISC problem has been addressed using different approaches in the literature (see [1, 3–7]). For example, Shao, Yang and Yu [1] used searchable encryption to fulfill private set intersection, in which public key encryption with multiple keywords search is used as the basic tool. Zhao and Luo proposed a two-party private set intersection protocol based on negative database [3]. The negative database is a new technique for preserving privacy, and it stores information in the complementary set of a traditional database. The security foundation of this technique is that reversing the negative database to recover the corresponding database is NP-hard. In the study of how to extract common sensitive information from encrypted sets, Liu et al [4] argued that existing methods are not suitable for cloud deployment. Hence, they designed the Encrypted Set Intersection Protocol (ESIP) that allows server and users to perform collaborative operations to obtain the correct set intersection with privacy-preserving. In the attempt to solve privacy-preserving intersection of regular languages instead of finite sets, Guanciale, Gurov and Laud [5] proposed an approach based on minimal deterministic finite automata. Wang, Zhu and Luo [6] proposed a scheme which allows any entity to publicly verify the correctness of set intersection query, without requiring any secret key. More recently in 2015, Thapa et al [7] used asymmetric social proximity to design different private matching protocols, designed to provide different privacy levels. We observe that the literature rarely requires a PISC solution to be both lightweight and flexible. In the big data era, lightweight and flexible are two critical properties for privacy-aware PISC [20, 21].

## 3 Problem Formulation

### 3.1 Network Model

There are three entities in the intersection set computation, namely: an entity A, an entity B, and a computation server C. Upon receiving the sets from A and B, C will compute and return the intersection sets to A and B. For privacy protection, A and B conduct their respective computations to conceal the original set prior to sending to C. C conducts the computation of PISC over the concealed sets and returns information on the intersection of original sets.

We denote the set from A to C, the set from B to C, the the intersection set from C to A and B, as *Set*_*A*_, *Set*_*B*_, and *Set*_*C*_, respectively. Thus, the basic logic flow in network model can be simplified as follows:

Msg1) *A* → *C* : *Set*_*A*_, where *x* → *y* : *z* denotes a message *z* being sent from entity *x* to entity *y*.

Msg2) *B* → *C* : *Set*_*B*_;

Msg3) *C* → *A* : *Set*_*C*_, *C* → *B* : *Set*_*C*_.

It is worth noting that *Set*_*A*_ and *Set*_*B*_ are concealed sets of the original sets, and *Set*_*C*_ can be redirected to the intersection of the original sets only at *A* and at *B* upon receipt.

### 3.2 Attack Model

We assume that the communication channels between entities and the server are protected by standard security mechanisms (e.g., encryption and integrity protection) at link layers (e.g., IEEE802.11i and CDMA); thus, attackers who can sniff packets are beyond the scope of this paper. In this paper, we focus on adversaries for privacy leakage at server side. In this context, we point out the following potential attacks, and we denote the adversary as *Adv*.

**Definition** Set Reveal Attack (*Adv*_*sra*_). The adversary reveals the privacy of entity A and entity B from observing *Set*_*A*_ and *Set*_*B*_. Roughly speaking, *H*(*x*) = *H*(*x*|*Set*_*A*_, *Set*_*B*_), where *H* is the entropy function and *x* is any information about A or B.

In other words, entity A and entity B present *Set*_*A*_ and *Set*_*B*_ to server C for discovering of the intersection members. As the server C is not entirely trustworthy, the presenting sets should not reveal the privacy of A or B. Thus, *Set*_*A*_ and *Set*_*B*_ must be transformed from the original sets to the concealed sets for further intersection computation. That is, *Set*_*A*_ and *Set*_*B*_ that are presented to the server C must not leak the privacy of entity A and entity B, respectively. In the tradition reductionist approach [22–24], the adversary we consider in this paper has an upper bound in computational capabilities (i.e., a probabilistic polynomial-time turing machine—PPTM).

*Adv*_*sra*_ captures the basic security notion, and the security can be achieved by some transformations guaranteeing computational secrecy. However, we observe that if sets presented to untrustworthy servers are “linkable”, it may also compromise the privacy of entity A (or B). This is because the adversary at the untrustworthy server can discover that entity A (or B) has conducted the same behavior more frequently than others, the interests or preference at entity A (or B) can then be inferred by the adversary (e.g., purchasing certain merchandise such as milk powder more frequently than others). We coin such an attack against the PISC as a Set Linkage Attack.

**Definition** Set Linkage Attack (*Adv*_*sla*_). The adversary can successfully guess that at least one item in *Set*_*A*_ (or *Set*_*B*_) in one round is the same as another entity in a previous round. Roughly speaking, $\text{Pr}\{\text{Find}\;x,x\in {Set}_{A}⋀x\in Set_{A}^{\prime }|$ Observing ${Set}_{A},Set_{A}^{\prime }\}>1/2$, where *Set*_*A*_ and ${Set}_{A}^{\prime }$ are in two distinct rounds.

For example, after the adversary observes *Set*_*A*_ in one round and ${Set}_{A}^{\prime }$ in another round, the adversary can link one member in *Set*_*A*_ to another member in ${Set}_{A}^{\prime }$. That is, the adversary can successfully guess there exists a same member in both *Set*_*A*_ and ${Set}_{A}^{\prime }$, which can compromise the privacy of that particular entity in some situations.

A more relaxed notion is to assume server C is semi-trustworthy (also known as honest-but-curious adversary). That is, the computation on server C for intersection set computation (mathematically) is trustworthy (i.e., correctly computed), but the adversary on server C may seek to infer the privacy of entity A and entity B by observing *Set*_*A*_ and *Set*_*B*_. Here, the computation functionality for intersection set has to be trustworthy as a prerequisite condition; otherwise, we will not be able to achieve privacy-aware intersection set computation.

Thus, the privacy requirements are stated as follows:

**Definition** $Pri_{PISC}^{Adv_{sra},Adv_{sla}}$. In the presence of *Adv*_*sra*_ and *Adv*_*sla*_ at a semi-trustworthy server C with a computational bound, the intersection set of *Set*_*A*_ and *Set*_*B*_ uploaded by entity A and entity B can be correctly computed without compromising the privacy of entity A and entity B. (Note that, the intersection set is for the original sets, rather than the concealed sets.)

### 3.3 Design Goals

The design of PISC should also consider flexibility, as *Set*_*A*_ and *Set*_*B*_ may not have the exact intersection set. In other words, we can also compute the rough intersection set even though *Set*_*A*_ and *Set*_*B*_ are not exactly or approximately equal. The flexibility will be particularly helpful in social network and e-commerce applications, as approximate intersection set is adequate for the required functionalities (e.g., identifying similar goods that may be of interest to an e-commerce user). Flexibility is also necessary when exact intersection set for the uploaded sets does not exist.

To achieve flexible PISC in a lightweight manner is also important, particularly due to the scale of data involved and the real-time nature of such computations. The lightweight computation in one time computation of PISC will significantly influence the scalability of the proposed method.

Therefore, the design goals are lightweight and flexible PISC.

## 4 Proposed Scheme - LiPISC

Table 1 outlines the notation used in the proposed Lightweight and Flexible PISC (LiPISC) scheme.

**Table 1:** Notation.

*Adv*	Adversary
*Adv*_*sra*_	Set Reveal Attack
*Adv*_*sla*_	Set Linkage Attack
*f*_*conceal*_	Concealing Function
*f*_*intersect*_	Intersection Set Computation Function
*f*_*reveal*_	Reveal Function
*Set*_*AC*_	Concealed Set from A
*Set*_*AO*_	Original Set from A
*Set*_*BC*_	Concealed Set from B
*Set*_*BO*_	Original Set from B
*Set*_*IC*_	“Intersection set” for Concealed Set
*Set*_*IAO*_	Indeed Intersection Set in Original Set from A
*Set*_*IBO*_	Indeed Intersection Set in Original Set from B

### 4.1 Abstract Model

LiPISC consists of the following functions:

1) *f*_*conceal*_. It takes as input the original set, *Set*_*AO*_, and outputs a concealed set, *Set*_*AC*_. That is,

$$\begin{array}{c} f_{conceal}:{Set}_{AO}\Rightarrow {Set}_{AC}, \end{array}$$

where *Set*_*A*_ ⇒ *Set*_*B*_ means that ∀*x* ∈ *Set*_*A*_, compute *y* = *f*_*conceal*_(*x*) and include *y* in *Set*_*B*_.

2) *f*_*intersect*_. It takes as input two sets, *Set*_*AC*_ and *Set*_*BC*_, and outputs a concealed intersection set, *Set*_*IC*_. That is,

$$\begin{array}{c} f_{intersect}:{Set}_{AC}\times {Set}_{BC}\Rightarrow {Set}_{IC}. \end{array}$$

3) *f*_*reveal*_. It takes as input a set, *Set*_*IC*_, and outputs an original intersection set, *Set*_*IO*_. That is,

$$\begin{array}{c} f_{reveal}:{Set}_{IC}\Rightarrow {Set}_{IAO},{Set}_{IC}\Rightarrow {Set}_{IBO}, \end{array}$$

where *Set*_*IO*_ = *Set*_*AO*_ ∧ *Set*_*BO*_, *Set*_*IAO*_ = *Set*_*IO*_ ∧ *Set*_*AO*_, *Set*_*IBO*_ = *Set*_*IO*_ ∧ *Set*_*BO*_. Certainly, *Set*_*IO*_ = *Set*_*IAO*_ = *Set*_*IBO*_.

Therefore, in LiPISC,

1. A and B conceal the original sets *Set*_*A*_ and *Set*_*B*_ using *f*_*conceal*_, and obtain concealed sets *Set*_*AC*_ and *Set*_*BC*_, respectively.
2. A and B respectively send concealed sets *Set*_*AC*_ and *Set*_*BC*_ to C.
3. C computes the intersection set *Set*_*IC*_ from the received concealed sets using *f*_*intersect*_. C then returns *Set*_*IC*_ to both A and B.
4. A finds the indeed intersection set (*Set*_*IAO*_) of the original sets (i.e., *Set*_*AO*_ ∧ *Set*_*BO*_) from *Set*_*IC*_ using *f*_*reveal*_. Similarly, B finds the indeed intersection set (*Set*_*IBO*_) of the original sets (i.e., *Set*_*AO*_ ∧ *Set*_*BO*_) from *Set*_*IC*_ using *f*_*reveal*_.

Thus, LiPISC can be formulated as an abstract model:

1) Entity A:

1.1) *f*_*conceal*_ : *Set*_*AO*_ ⇒ *Set*_*AC*_;

1.2) *A* → *C* : *Set*_*AC*_.

2) Entity B:

2.1) *f*_*conceal*_ : *Set*_*BO*_ ⇒ *Set*_*BC*_;

2.2) *B* → *C* : *Set*_*BC*_.

3) Server C:

3.1) *f*_*intersect*_ : *Set*_*AC*_ × *Set*_*BC*_ ⇒ *Set*_*IC*_;

3.2) *C* → *A* : *Set*_*IC*_;

3.3) *C* → *B* : *Set*_*IC*_.

4)

Entity A:

4.1) *f*_*reveal*_ : *Set*_*IC*_ ⇒ *Set*_*IAO*_;

Entity B:

4.2) *f*_*reveal*_ : *Set*_*IC*_ ⇒ *Set*_*IBO*_.

The requirement is as follows: *f*_*conceal*_.

**Proposition 4.1** *f*_*conceal*_ *should achieve the functionality to defend against* *Adv*_*sra*_ *and* *Adv*_*sla*_.

**Proof** *f*_*conceal*_ is the only transformation of the original set before the observation of the adversary. After this transformation, the adversary at C can observe the transformation result of *f*_*conceal*_. Thus, *f*_*conceal*_ should defend against *Adv*_*sra*_ and *Adv*_*sla*_. ▫

*f*_*conceal*_ should make it possible to find the corresponding *f*_*intersect*_ and *f*_*reveal*_. That is, corresponding *f*_*intersect*_ and *f*_*reveal*_ should be found easily and should perform efficiently after the transformation of *f*_*conceal*_. Thus, *f*_*conceal*_ is the most important of three functions. Besides, these three functions must be lightweight in terms of computation. Therefore, finding proper *f*_*conceal*_ has the highest priority in searches.

### 4.2 Basic Construction

The basic construction is described as follows:

1) Entity A:

1.1) *f*_*conceal*_ : *Set*_*AO*_ ⇒ *Set*_*AC*_. Let $f_{conceal}=Hash\left(\cdot \right):{\left\{0,1\right\}}^{m}\to {\left\{0,1\right\}}^{n},m,n\in N$; That is, *Set*_*AC*_ ⇐ *Hash*(*x* ∈ *Set*_*AO*_), where *Hash*(⋅) could be a cryptographic hash function. In other words, *Set*_*AC*_ = {*y*|*y* = *Hash*(*x*), ∀*x* ∈ *Set*_*AO*_}.

1.2) *A* → *C* : *ID*[*i*], *Set*_*AC*_[*i*], where *i* = 1, …, |*Set*_*AC*_|; |∗| returns the number of items in the set ∗; *ID*[*i*] is a temporary sequence number for *i*-th item in *Set*_*AC*_, which is denoted as *Set*_*AC*_[*i*];

2) Entity B:

2.1) *B* : *f*_*conceal*_ : *Set*_*BO*_ ⇒ *Set*_*BC*_; Similar to 1.1), let *f*_*conceal*_ = *Hash*(⋅); *Set*_*BC*_ ⇐ *Hash*(*Set*_*BO*_), where *Hash*(⋅) could be a one-way cryptographic hash function. In other words, *Set*_*BC*_ = *Hash*(*x* ∈ *Set*_*BO*_) = {*y*|*y* = *Hash*(*x*), ∀*x* ∈ *Set*_*BO*_}.

2.2) *B* → *C* : *ID*[*i*], *Set*_*BC*_[*i*], where *i* = 1, …, |*Set*_*BC*_|; *ID*[*i*] is a temporary sequence number for *i*-th item in *Set*_*BC*_, which is denoted as *Set*_*BC*_[*i*];

3) Server C:

3.1) *f*_*intersect*_ : *Set*_*AC*_ ∧ *Set*_*BC*_ ⇒ *Set*_*IC*_;

3.2) *C* → *A* : *ID*[*i*], where *Set*_*AC*_[*i*] ∈ *Set*_*IC*_, *i* ∈ [1, |*Set*_*AC*_|]; The total number of *i* is |*Set*_*IC*_|; That is, |*ID*[*i*]| = |*Set*_*IC*_|;

3.3) *C* → *B* : *ID*[*j*], where *Set*_*BC*_[*j*] ∈ *Set*_*IC*_, *j* ∈ [1, |*Set*_*BC*_|]; The total number of *j* is |*Set*_*IC*_|; That is, |*ID*[*j*]| = |*Set*_*IC*_|;

4)

Entity A:

4.1) *f*_*reveal*_ : *ID*[*i*]⇒*Set*_*IAO*_; As *A* has computed *Set*_*AC*_ ⇐ *Hash*(*Set*_*AO*_) before, *A* can retrieve corresponding *i*-th item in *Set*_*AO*_ that will compose final *Set*_*IAO*_;

Entity B:

4.2) *f*_*reveal*_ : *ID*[*j*]⇒*Set*_*IBO*_; As *B* has computed *Set*_*BC*_ ⇐ *Hash*(*Set*_*BO*_) before, *A* can retrieve corresponding *j*-th item in *Set*_*BO*_ that will compose final *Set*_*IBO*_;

Remarks

1) *f*_*conceal*_ is pre-deployed or negotiated in advance at A and B, or distributed by C instantly and publicly. Even though *f*_*conceal*_ is publicly known, *Adv*_*sra*_ is still defended against due to the cryptographic properties of *Hash*(⋅).

2) Note that *Hash*(⋅) could be any function with dedicated requirements, although a computationally efficient cryptographic hash is usually preferred. Next, we will define the one-way and collision resistant requirements.

**Proposition 4.2** $f_{conceal}:{\left\{0,1\right\}}^{m}\to {\left\{0,1\right\}}^{n},m\geq n\in N$ *should be one-way*.

**Proof** Due to 4.1, *f*_*conceal*_ should have functionality of defending against *Adv*_*sra*_. *H*(*x*|*f*_*conceal*_(*x*)) = *H*(*x*) for PPTM adversary. *Pr*{*x*|*y* = *f*_*conceal*_(*x*)} = 1/2^*n*^ ∗ 1/2^*m*−*n*^ = 1/2^*m*^ = *negl*(*m*) should be negligible when *m* is sufficient large. *negl*(*m*) is a negligible function in *m*. Given *y* compute *x* is hard; thus, *f*_*conceal*_ must be one-way. ▫

**Proposition 4.3** *f*_*conceal*_ *should be collision resistant*.

**Proof** *f*_*conceal*_ should be collision resistant, which is not for security but for the soundness of resulting intersection set returned from C. If so, the probability will be negligible that *y*_1_ ∈ *Set*_*AC*_ and *y*_2_ ∈ *Set*_*BC*_ are equal but *x*_1_ ∈ *Set*_*AO*_ and *x*_2_ ∈ *Set*_*BO*_ are not equal. ▫

Note that it is non-trivial to be aware that *f*_*conceal*_ is not second pre-image resistant, because server C has no idea of the pre-image of *Set*_*AC*_ as well as *Set*_*BC*_.

**Proposition 4.4** *The false probability of basic construction for PISC is* 1/2^*m*−*n*^. *That is*, Pr{*x*_1_ ∈ *Set*_*AO*_ ≠ *x*_2_ ∈ *Set*_*BO*_|*y* = *f*_*conceal*_(*x*_1_) ∈ *Set*_*AC*_, *y* = *f*_*conceal*_(*x*_2_) ∈ *Set*_*BC*_} = 1/2^*m*−*n*^.

**Proof** There are 1/2^*m*−*n*^ *x* ∈ *Set*_*AO*_ mapping into the same *y* ∈ *Set*_*AC*_. Thus, even *y* is the same *x* is different. The false probability of basic construction is 1/2^*m*−*n*^. ▫

3) Only last *k*, *k* < |*Hash*(⋅)| = *n* bits can be selected in *Hash*(⋅) during comparison to further improve the efficiency, but it may induce extra false members in intersection set with false probability (namely, 1/2^*m*−*k*^).

The privacy strength for the basic construction is as follows:

**Proposition 4.5** *Basic construction can defend against* *Adv*_*sra*_ *but not* *Adv*_*sla*_.

**Proof** As *f*_*conceal*_ is one-way, the adversary can reveal neither *Set*_*AO*_ from *Set*_*AC*_ nor *Set*_*BO*_ from *Set*_*BC*_. Thus, *Adv*_*sra*_ is defended against. However, the same *y* ∈ *Set*_*AC*_(*Set*_*BC*_) in different rounds can be linked to the same *x* ∈ *Set*_*AO*_(*Set*_*BO*_). Thus, *Adv*_*sla*_ is not defended against. ▫

### 4.3 An Enhanced Basic Construction

To defend against *Adv*_*sla*_, we propose the following enhancement by adding a random number to be used only once (i.e., nonce) in *f*_*conceal*_ to make it unlinkable when the adversary observes *Set*_*AC*_ (or *Set*_*BC*_). More specifically, the enhancement is at steps 1.1) and 2.1) with the addition of the nonce, respectively. The enhanced steps 1.1) and 2.1) are as follows:

1.1) *f*_*conceal*_ : *Set*_*AO*_ ⇒ *Set*_*AC*_. Let *f*_*conceal*_ = *Hash*(⋅); That is, *Set*_*AC*_ ⇐ *H*(*nonce*_*A*_||*Set*_*AO*_), where *Hash*(⋅) could be a cryptographic hash function; *nonce* is a number used once. In other words, ∀*x* ∈ *Set*_*AO*_, *y* ⇐ *Hash*(*nonce*_*A*_||*x*), *y* ∈ *Set*_*AC*_.

2.1) *f*_*conceal*_ : *Set*_*BO*_ ⇒ *Set*_*BC*_; Similar to 1.1), let *f*_*conceal*_ = *Hash*(⋅); *Set*_*BC*_ ⇐ *Hash*(*nonce*_*B*_||*Set*_*BO*_), where *H*(⋅) is a cryptographic hash function with one-wayness. In other words, ∀*x* ∈ *Set*_*BO*_, *y* ⇐ *Hash*(*nonce*_*B*_||*x*), *y* ∈ *Set*_*BC*_.

Remarks

1) *Nonce*_*A*_ and *Nonce*_*B*_ could be timestamps, in which A and B need to be synchronized in advance.

2) *Nonce*_*A*_ and *Nonce*_*B*_ could be a value from a pseudorandom number generator, which is generated from a shared secret key (seed) and synchronized at A and B. That is, *Nonce* = *PRNG*(*k*), where *k* is a shared secret key between A and B (e.g., generated using a key establishment protocol [25]); *PRNG*(⋅) is a pseudorandom number generator.

3) *Nonce*_*A*_ and *Nonce*_*B*_ could be a synchronized counter.

**Proposition 4.6** *The enhanced basic construction can defend against* *Adv*_*sra*_ *and* *Adv*_*sla*_.

**Proof** The enhanced basic construction can defend against *Adv*_*sra*_ inherently due to basic construction. The discussion, thus, only concentrates on *Adv*_*sla*_. As *Nonce*_*A*_ and *Nonce*_*B*_ vary in each round, the same *y* ∈ *Set*_*AC*_(*Set*_*BC*_) in different rounds usually cannot be identified by the adversary due to the collision resistance property of *f*_*conceal*_. Thus, the linkage will not be drawn and *Adv*_*sla*_ is defended against. ▫

### 4.4 Advanced Construction with Rough Intersection for Flexibility

In a real-world deployment, such as on-line social networks and e-commerce websites, entity A and entity B are unlikely to have the exact number or same members in the submitted sets. Although exact intersection of *Set*_*AO*_ and *Set*_*BO*_ dose not exist, server C is still required to return approximate intersection of *Set*_*AO*_ and *Set*_*BO*_. In this scenario, server C has to recommend the most approximate intersection of *Set*_*AO*_ and *Set*_*BO*_ from *Set*_*AC*_ and *Set*_*BC*_. The basic construction and the enhanced basic construction are not able to address such a situation. Thus, we propose an advanced method that can satisfy this requirement.

Intuitively, if *Hash*(⋅) is “fuzzy” in that ‖*x*_1_ − *x*_2_‖ ∈ Δ ⇒ ‖*y*_1_ − *y*_2_‖ ∈ Δ, *y*_1_ = *Hash*(*x*_1_), *y*_2_ = *Hash*(*x*_2_), the rough set will be returned. Here, ‖⋅‖ returns either the absolute value or the hamming distance. However, this fuzzy property may compromise the one-wayness.

The improvements are due to the following: at *f*_*conceal*_, *Hash*(⋅) is replaced by $G\left(\cdot \right):g^{x}\;\mathrm{mod}\;p$, where *p* is a sufficient large prime, and at *f*_*intersect*_, the comparison is changed into computation of $\left(g^{x_{1}}\right)/\left(g^{x_{2}}\right)\approx g^{\delta }$. More specifically, the method is described as follows:

1) Entity A:

1.1) *A* : *f*_*conceal*_ : *Set*_*AO*_ ⇒ *Set*_*AC*_. Let $f_{conceal}=g^{x}\;\mathrm{mod}\;p$, where *x* ∈ *Set*_*AO*_; *p* is a large prime. That is, *Set*_*AC*_ ⇐ *G*(*Set*_*AO*_), where *G*(*Set*_∗_) means to compute *f*_*conceal*_(*x*) for ∀*x* ∈ *Set*_∗_. In other words, ${Set}_{AC}=\left\{y|\forall x\in {Set}_{AO},y=g^{x}\;\mathrm{mod}\;p\right\}$.

1.2) *A* → *C* : *ID*[*i*], *Set*_*AC*_[*i*], where *i* = 1, …, |*Set*_*AC*_|; |∗| returns the number of items in the set ∗; *ID*[*i*] is a temporary sequence number for *i*-th item in *Set*_*AC*_, which is denoted as *Set*_*AC*_[*i*];

2) Entity B:

2.1) *B* : *f*_*conceal*_ : *Set*_*BO*_ ⇒ *Set*_*BC*_; Similar to 1.1), let $f_{conceal}=g^{x}\;\mathrm{mod}\;p$, where *x* ∈ *Set*_*BO*_; That is, *Set*_*BC*_ ⇐ *G*(*Set*_*BO*_). In other words, ${Set}_{BC}=\left\{y|\forall x\in {Set}_{BO},y=g^{x}\;\mathrm{mod}\;p\right\}$.

2.2) *B* → *C* : *ID*[*i*], *Set*_*BC*_[*i*], where *i* = 1, …, |*Set*_*BC*_|; *ID*[*i*] is a temporary sequence number for *i*-th item in *Set*_*BC*_, which is denoted as *Set*_*BC*_[*i*];

3) Server C:

3.1) *f*_*intersect*_ : ∀*Set*_*AC*_[*i*] ∈ *Set*_*AC*_, *Set*_*BC*_[*j*] ∈ *Set*_*BC*_, if *Set*_*AC*_[*i*]/*Set*_*BC*_[*j*] < *g*^*δ*^ < *p*, let *Set*_*AC*_[*i*]∨*Set*_*BC*_[*j*]⇒*Set*_*IC*_;

3.2) *C* → *A* : *ID*[*i*], where *Set*_*AC*_[*i*] ∈ *Set*_*IC*_, *i* ∈ [1, |*Set*_*AC*_|]; The total number of *i* is |*Set*_*IC*_|/2; That is, |*ID*[*i*]| = |*Set*_*IC*_|/2;

3.3) *C* → *B* : *ID*[*j*], where *Set*_*BC*_[*j*] ∈ *Set*_*IC*_, *j* ∈ [1, |*Set*_*BC*_|]; The total number of *j* is |*Set*_*IC*_|/2; That is, |*ID*[*j*]| = |*Set*_*IC*_|/2;

4)

Entity A:

4.1) *f*_*reveal*_ : *ID*[*i*]⇒*Set*_*IAO*_; As *A* computes *Set*_*AC*_ ⇐ *G*(*Set*_*AO*_), *A* can reveal corresponding *i*-th item in *Set*_*AO*_ that consists final *Set*_*IAO*_;

Entity B:

4.2) *f*_*reveal*_ : *ID*[*j*]⇒*Set*_*IBO*_; As *B* computes *Set*_*BC*_ ⇐ *G*(*Set*_*BO*_), *A* can reveal corresponding *j*-th item in *Set*_*BO*_ that consists final *Set*_*IBO*_;

Remarks

1) To reduce the computation overhead at entity A and entity B, in computing *G*(⋅), A and B can only compute the last *k* bits of *x*, *y*, ∀*x* ∈ *Set*_*AO*_ and ∀*y* ∈ *Set*_*BO*_. log_2_ *x* = {0,1}^*m*^ = {0,1}^*m* − *k*^‖{0,1}^*k*^, log_2_ *y* = {0,1}^*n*^ = {0,1}^*n* − *k*^‖{0,1}^*k*^. It will not compromise the soundness of PISC if *k* is properly chosen. We state the chosen method in the following proposition.

**Proposition 4.7** *If the absolute gap value between* *x* ∈ *Set*_*AO*_ *and* *y* ∈ *Set*_*BO*_ *is* ‖*x* − *y*‖, *we let* *k* = log_2_ log_*g*_‖*x* − *y*‖.

**Proof** *x* ∈ *Set*_*AO*_, *g*^*x*^ mod *p* ∈ *Set*_*AC*_. *x* ∈ *Set*_*BO*_, *g*^*x*^ mod *p* ∈ *Set*_*BC*_. $\frac{g^{x}}{g^{y}}$ can be computed via $\frac{g^{m+(x-m)}}{g^{m+(y-m)}},$ where log_*g*_(*x* − *m*) < 2^*k*^ and log_*g*_(*y* − *m*) < 2^*k*^. Thus, only computing the last *k* bits of *x*, *y* will not result in any difference for $\frac{g^{x}}{g^{y}}$ at server C. ▫

2) *δ* is a system parameter, which measures the approximate strength of the intersection set. More specifically, we state it formally in the following proposition.

**Proposition 4.8** *If the absolute gap value between* *x* ∈ *Set*_*AO*_ *and* *y* ∈ *Set*_*BO*_ *is* ‖*x* − *y*‖, *the intersection set produced by PISC will have* ‖*x* − *y*‖ ≤ *δ*.

**Proof** *x* ∈ *Set*_*AO*_, $g^{x}\;\mathrm{mod}\;p\in {Set}_{AC}$. *y* ∈ *Set*_*BO*_, $g^{y}\;\mathrm{mod}\;p\in {Set}_{BC}$. Server C selects intersection set by $\frac{g^{x}}{g^{y}}<g^{\delta },$ thus ‖*x* − *y*‖ ≤ *δ* + *kϕ*(*p*) = *δ* + *k*(*p* − 1), where *ϕ*(⋅) is Euler function, and k∈N. *p* − 1 >> *δ* and *x* ≤ *p* − 1, *y* ≤ *p* − 1, thus ‖*x* − *y*‖ ≤ *δ*. ▫

From above two propositions, we can choose *k* = log_2_ log_*g*_‖*x* − *y*‖ = log_2_ log_*g*_ *δ* bits at rear of log_2_ *x*, ∀*x* ∈ *Set*_*AO*_ or log_2_ *y*, ∀*y* ∈ *Set*_*BO*_ to compute *Set*_*AC*_ and *Set*_*BC*_, respectively.

3) The computation overhead at server C is module exponential computation (for computing $g^{\delta }\;\mathrm{mod}\;p$) and one time division (for computing *Set*_*AC*_[*i*]/*Set*_*BC*_[*j*]). The computation overhead at an entity is module exponential computation (for computing $g^{k}\;\mathrm{mod}\;p$ and *k* is at most log_2_ log_*g*_ *δ* bits).

**Proposition 4.9** *The advanced construction can defend against* *Adv*_*sra*_, *but not* *Adv*_*sla*_.

**Proof** As *f*_*conceal*_ is one-way due to the underlying discrete logarithm problem, *Adv*_*sra*_ is defended against. As *f*_*conceal*_ is a deterministic function, the same image will link to the same pre-image; thus, *Adv*_*sla*_ cannot be defended against. ▫

**Proposition 4.10** *In the advanced construction*, *f*_*conceal*_ *is collision resistant*.

**Proof** The aim is to prove that it is difficult to find *x* ≠ *y* such at *g*^*x*^ ≡ *g*^*y*^ mod *p*. That is, it is difficult to find *x* ≠ *y* such that *g*^*x*−*y*^ mod *p* = 1. If *g*^*x*−*y*^ mod *p* = 1, *x* − *y* mod *p* − 1 = 0 by Fermat’s theorem. As *x* − *y* < *δ* and *p* is a big prime, it is computational infeasible for x-ymodp-1=0. ▫

**Proposition 4.11** *The advanced construction is sound*.

**Proof** It is concluded from the previous propositions. *f*_*conceal*_ is one-way, collision resistant, and the returned intersection sets are indeed the approximate members. Thus, the advanced construction is sound. ▫

Finally, to defend against *Adv*_*sla*_, we propose an enhancement method for the advanced construction by adding a nonce in *f*_*conceal*_. More specifically, the enhancement is at Steps 1.1) and Step 2.1) by multiplying the nonce, respectively:

1.1). *f*_*conceal*_ = *nonce* ∗ *g*^*x*^ mod *p*, where *x* ∈ *Set*_*AO*_; *p* is a large prime. That is, *Set*_*AC*_ ⇐ *G*(*Set*_*AO*_), where *G*(*Set*_∗_) means to compute *f*_*conceal*_(*x*) for ∀*x* ∈ *Set*_∗_. In other words, ${Set}_{AC}=\left\{y|\forall x\in {Set}_{AO},y=nonce*g^{x}\;\mathrm{mod}\;p\right\}.$

2.1) *f*_*conceal*_ = *nonce* ∗ *g*^*x*^ mod *p*, where *x* ∈ *Set*_*BO*_; That is, *Set*_*BC*_ ⇐ *G*(*Set*_*BO*_).

The discussion on *nonce* is similar to the aforementioned remarks in the last section. The security proof after the inclusion of *nonce* is similar to Proposition 4.6. Until now, we reach the final proposed version of PISC that is the enhancement of the advanced construction. This incremental presentation helps for better understanding and smoothly remembering.

Potential Applications and Further Discussions

1) Example I. In social networks for recommending prospective friends, both users B and C are user A’s friends; thus, the intersection set of B’s friends and C’s friends may also be A’s friends. The service provider needs to perform PISC of two inputs—B’s friends and C’s friends, and those inputs should be concealed for privacy protection.

2) Example II. For recommending potentially goods of interest in an e-commence website, B’s purchase history and C’s purchase history have overlapping items with A’s purchase history (i.e., A, B, C have similar purchasing habits and preferences); thus, the intersection set of user B’s purchases and C’s purchases may also interest A. The service provider needs to perform PISC of two concealed inputs—B’s purchase history and C’s purchase history. Since we require that the purchase history of both B and C to be unlinkable, the service provider is unable to guess whether the same item exists in B’s or C’s purchase history.

3) As in LiPISC, A and B have to establish a shared nonce, which may require synchronization at both A and B. Alternatively, nonce can also be distributed by servers which does not break the security of the scheme as the servers will not know the nonce due to the one-wayness of *f*_*conceal*_.

4) As stated in Section 3 (i.e., Problem Formulation), the adversary (trust) model in this paper only assumes an adversary at a central server. That is, entity A and entity B for PISC is trustworthy. Nonetheless, even though A (or B) could be un-trustworthy, A (or B) can only reveal the intersection set with B (or A) by random guess due to the one-wayness of *f*_*conceal*_. The probability of successful guess is negligible due to the selection of *f*_*conceal*_.

5) The gap between numeric distance represents the numeric deviation of inputting values, and the gap between Hamming distance represents the property deviation. The former gap can be computed from the latter gap if the major difference comes from the bits at the end, and the latter gap can also be estimated from the former gap. In this paper, we focus on the numeric distance, which is more general than the Hamming gap. In addition, the proposed method is suitable for both two situations, as here only rough intersection set computation is required.

6) The proposed scheme outperforms related work in sever aspects as follows: It is lightweight as only raw discrete logarithm computation is involved instead of cryptographic encryption. It can compute rough intersection set that is flexible in realistic. It tackles set linkage attack that has not been carefully explored in the literatures.

## 5 Conclusion

In this paper, we introduced two new attacks against PISC, which we coined as set reveal attack and set linkage attack. We then proposed a lightweight and flexible PISC (LiPISC), which achieves approximate intersection set computation and rough intersection set computation in a lightweight manner. We then proved the security of LiPISC.

Future work includes deploying the proposed scheme in a real-world application, such as e-commerce website, with the aims of refining and validating the scheme.
